# Development of an optimized colorimetric RT-LAMP for SARS-CoV-2 assay with enhanced procedure controls for remote diagnostics

**DOI:** 10.1038/s41598-022-25872-1

**Published:** 2022-12-11

**Authors:** Bruna Winkert Raddatz, Edson Yu Sin Kim, Louise Matiê Imamura, Gisleine Jarenko Steil, Erika Bergamo Santiago, Santiago Pedro Timm Soares, Victor Henrique Alves Ribeiro, Bernardo Montesanti Machado de Almeida, Sergio Renato Rogal, Marcus Vinícius Mazega Figueredo

**Affiliations:** Hilab, Rua José Altair Possebom, 800 - CIC, Curitiba, PR 81270-185 Brazil

**Keywords:** Diagnosis, Viral infection

## Abstract

The coronavirus pandemic accentuated the need for molecular diagnostic tests. A technique highly used to this end is the Polymerase Chain Reaction (PCR)—a sensitive and specific technique commonly used as the gold standard for molecular diagnostics. However, it demands highly trained personnel and high-maintenance equipment and is relatively time-consuming. An alternative is the Loop-Mediated Isothermal Amplification (LAMP) technique, which doesn’t need sample purification or expensive equipment, and is similar to PCR when compared in sensitivity and specificity. In this paper, we developed an optimized colorimetric Reverse Transcriptase Loop-Mediated Isothermal Amplification (RT-LAMP) Point-of-Care test using a portable device to diagnose COVID-19. Variables such as concentration of primers, magnesium sulfate, betaine, hydrochloride guanidine, *Bst*, and temperature of the reactions were tested. We also created a pipetting quality control system—using a combination of dyes—to avoid false negatives due to a lack of samples added to the reaction test tube. Mineral oil was incorporated in the composition of the RT-LAMP reactions to avoid evaporation when a heating lid isn't available. The final RT-LAMP test is tenfold more sensitive when compared to the WarmStart Colorimetric Master mix from New England Biolabs with a sensitivity of 5 copies per μL.

## Introduction

In December 2019, the world witnessed the outcome of a new coronavirus, named Severe Acute Respiratory Syndrome Coronavirus 2, or SARS-CoV-2. The first case of the infection by SARS-CoV-2 was reported in Wuhan, China, and soon it spread throughout the whole globe, being notified as a new pandemic in March 2020^1^. The outbreak of the pandemic revealed the global need for molecular biology tests to identify, isolate and treat infected people as means to control the spread of the disease.

Reverse Transcriptase Polymerase Chain Reaction (RT-PCR)^2^ is commonly known as the gold standard technique for molecular testing of RNA viruses, such as SARS-CoV-2. RT-PCR is a technique with high sensitivity and specificity, but it requires high-cost and high-maintenance equipment, extensive sample purification steps, and highly trained personnel. With the rapid increase of infected people, the lack of reagents, inputs, personnel, and equipment for molecular testing became evident, and the world experienced a shortage of laboratory resources to meet the demand.

Loop-Mediated Isothermal Amplification (LAMP) was first described by Notomi and his colleagues in 2000^3^ as a nucleic acid amplification technique with high specificity and efficiency, capable of being performed under isothermal conditions. It uses a strand-displacement DNA polymerase (deprived of 5′ → 3′ exonuclease activity) and a set of two to three pairs of primers designed to create loops in the synthesized single-strand DNA. Each time the polymerase starts a new amplification, it releases the previously paired DNA strand, which forms a loop structure that contains new binding sites for the polymerase, thus creating a self-propagate amplification. The final products of the reaction are cauliflower-like structures with multiple loops formed by annealing between alternately inverted repeats of the target^3^. As well as in PCR, LAMP reactions can also be coupled with a Reverse Transcriptase enzyme to perform amplification of RNA molecules.

The high degree of DNA synthesis occurring in LAMP reactions allows the visual detection of a positive reaction. Whenever the DNA polymerase incorporates a dNTP in the newly synthesized DNA strand, it releases a pyrophosphate and a proton molecule as a by-product. The pyrophosphate, in the presence of Mg^2+^ ions, precipitates as magnesium pyrophosphate changing the turbidity of the reaction. The proton released decreases the pH of the reaction, which can be detected by using a pH-sensitive colorimetric dye such as phenol red and bromothymol blue^4,5^.

Isothermal Colorimetric Nucleic Acid Amplification Tests (NAATs) have played a central role in the response to the COVID-19 pandemic. Beyond the fact that these do not require a thermocycler, sample processing is also simplified^6^, and results can be determined with the naked eye. These techniques have been applied to Point-of-Care and treatment (POCT) settings with success, with digital sensors aiding reaction control and analysis. Beyond the simple setup, colorimetric RT-LAMP can also be used in POCT diagnostic tests with RGB (Red, Green, Blue) detection for increased accuracy while excluding the need for expansive filters and detectors. Analysis of time course data can yield amplification curves similar to fluorescent systems^7^.

Here we perform a systematic development of a colorimetric RT-LAMP assay targeting SARS-CoV-2, optimized for use in the Hilab® Molecular POCT device, including reagent concentration fine-tuning, quality control procedures for the operator, evaporation management, and stability.

## Results

### DoE analysis of RT-LAMP formulation

**Figure 1:**
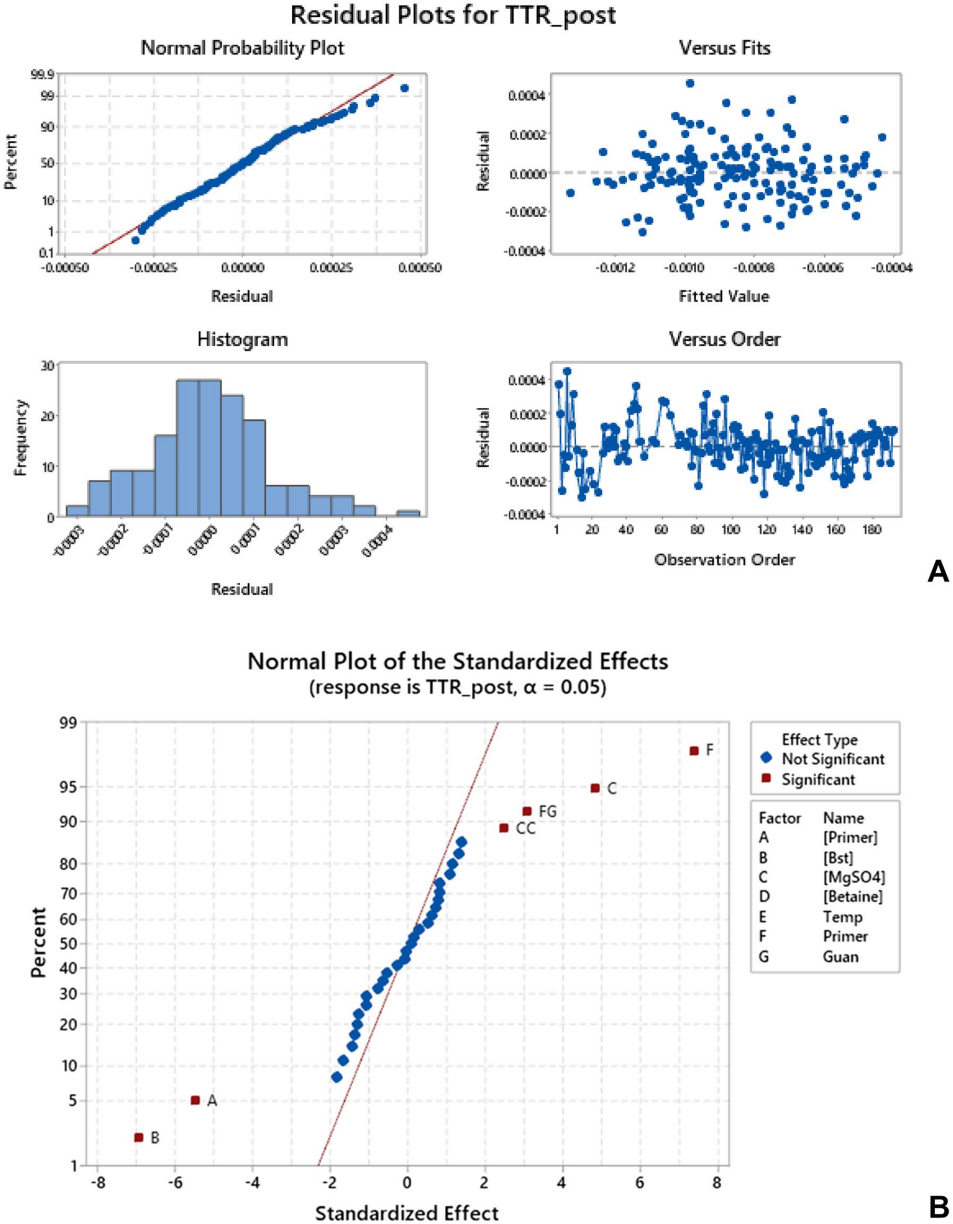
Analysis of the experiment results aiming to minimize the Time To Reaction (TTR) response of positive reactions, obtained with the software Minitab. In (**A**) Residual plots. The normal probability plot represents that the residuals analyzed are normally distributed. The histogram represents the distribution of the residuals. The residual versus fits plot shows that the residuals are randomly distributed, and the residual versus order plot indicates the residuals in the order that the data were collected, showing that the residuals are independent since there are no patterns in the observation order. (**B**) Normal probability plot with α = 0.05, where the significant factors, in order, were: primer sequence (F), *Bst* concentration (B), primer concentration (A), magnesium sulfate concentration (C), and the combination of primer sequence and adjust of hydrochloride guanidine pH (FG).

**Figure 2:**
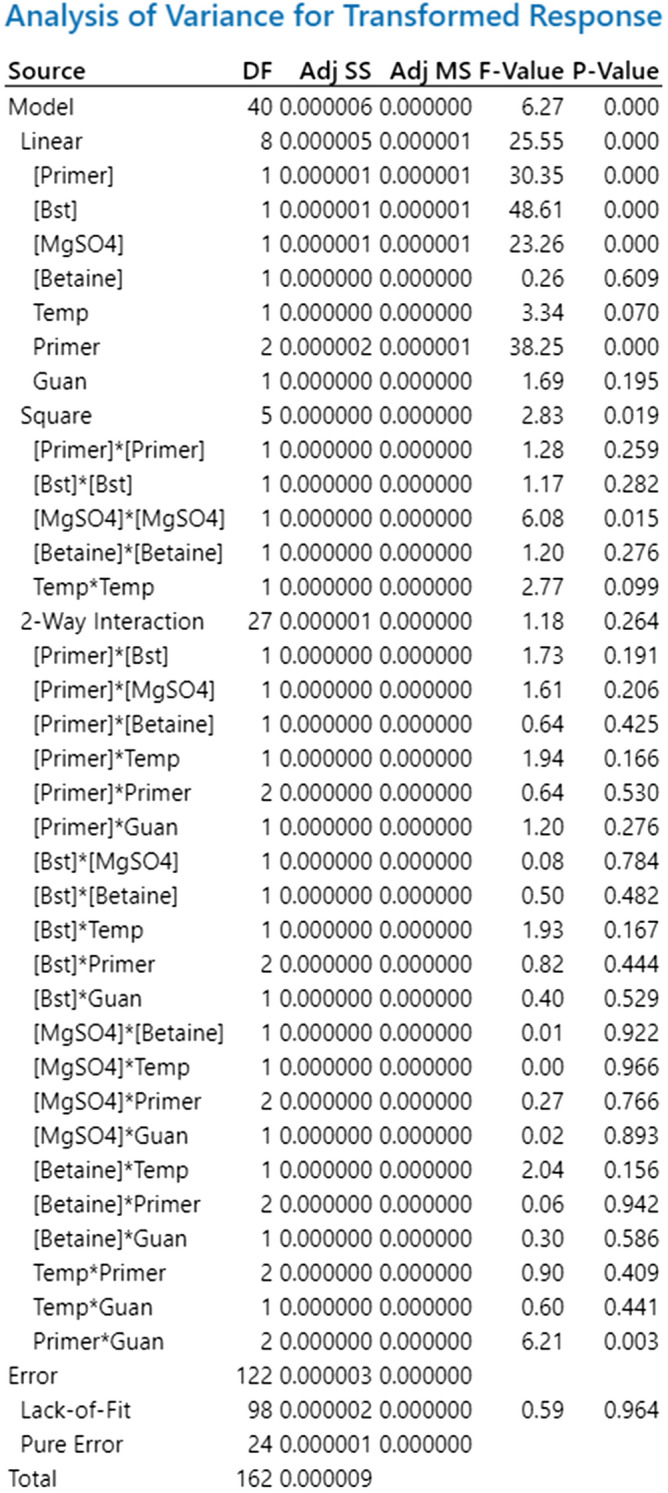
ANOVA of the TTR response obtained from the software Minitab with the tested factors and the respective *P*-value. The factors such as primer concentration ([Primer]), *Bst* concentration ([*Bst*]), magnesium sulfate concentration ([MgSO4]), and primer sequence (Primer) were statistically significant, *p* < 0.05.

**Figure 3:**
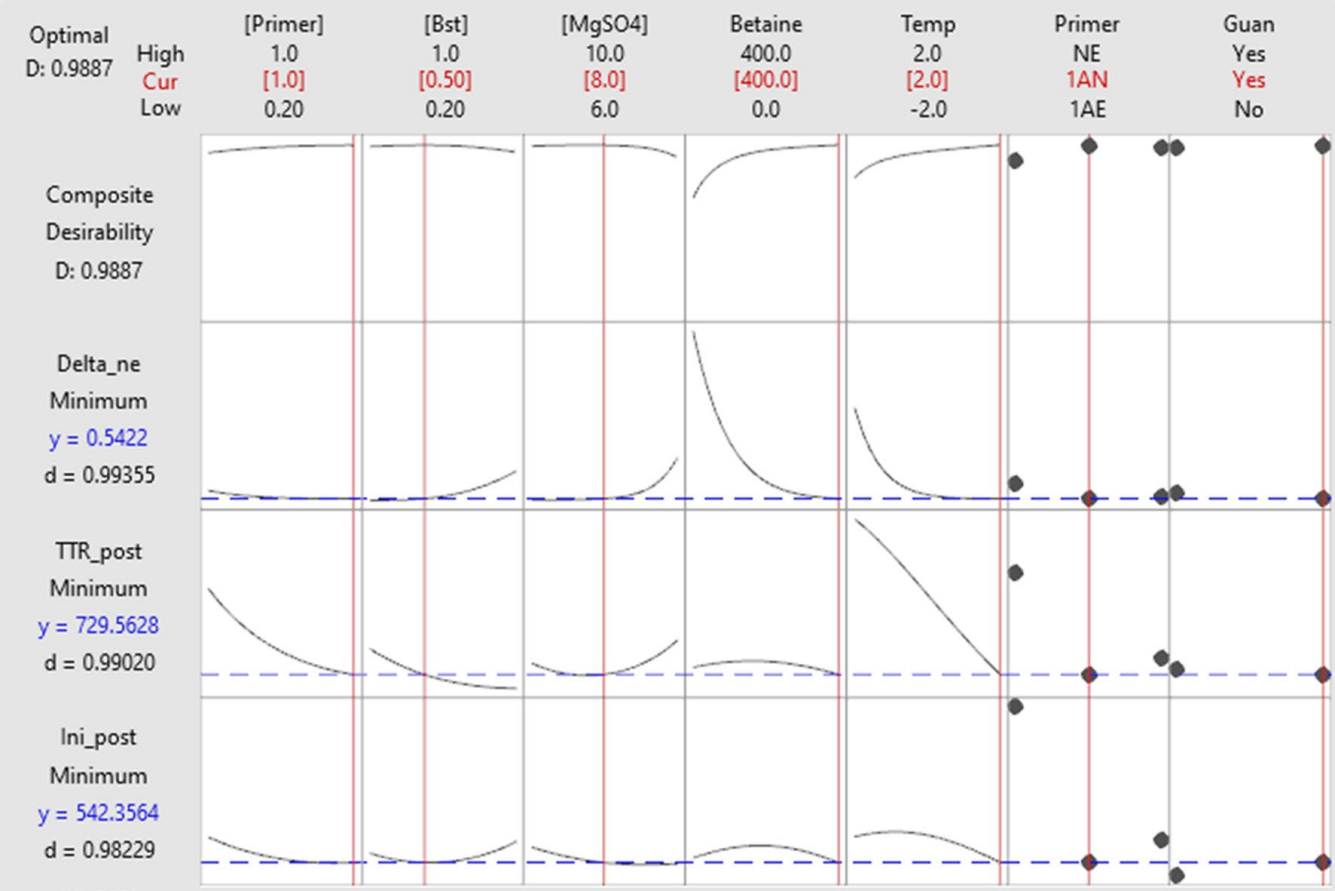
Response optimization obtained from Minitab by minimizing the initial positivity (Ini_post) and TTR (TTR_post) of positive reactions, and minimizing the Nmax (Color Delta) of negative reactions with the desirability of 0.989. To achieve the optimal response the final model was primer at onefold concentration, *Bst* at 0.4 U/μL (equivalent to 50%), magnesium sulfate at 8 mM, betaine at 400 mM, primers targeting the N and Orf1ab sequence, and temperature of 67 °C.

The complete experimental matrix was composed of 192 different conditions (Table S1). A model was defined for each response parameter, for both positive and negative reactions, and factors were maintained or removed based on a Pareto Chart of the standardized effects, with alpha (α) = 0.05. We performed the experiments with a single replicate, and outliers were manually removed based on the curve shape and expected results. Figure 1 illustrates the analysis of the experiment results, including residue profiles and normal probability plots. Model fit was deemed acceptable due to the profile of residue distribution. The ANOVA of the models can be found in Fig. 2, and Fig. 3 illustrates the response optimization aiming to minimize the initial time of amplification (Xt) and Time To Reaction (TTR)—the time to achieve 50% of the amplification delta—of positive reactions and minimize the Nmax (Delta of the color change between the initial and the final color of the reaction) of negative reactions.

Modeling the results of the TTR response of positive reactions yields primer sequence, enzyme concentration, magnesium sulfate, and primer concentration as significant factors, in this order. The modeling for manual determination of amplification start (Xt) and inflection point using non-linear regression was essentially the same. Optimization to minimize the modeled response variables was performed and factorial and desirability plots were used to determine ranges of interest for the analyzed factors. Primer concentration at onefold (the maximum value tested) was found to be optimal, with lower concentrations causing an increase in the TTR values. Higher temperatures had a positive effect on reaction times. Primer sequence type was worse for the NE pair, and roughly the same for the remaining two combinations, while guanidine hydrochloride (Gu-HCl) stock solution pH adjustment seems to have little to no effect on the evaluated responses.

The model for the Nmax of negative control reactions was also accepted, with R^2^ of 67.28%. Factorial plots of the model indicate a propensity of non-specific amplification for increased concentrations of polymerase and magnesium sulfate. An inverse relationship between betaine concentration is observed. Lower temperatures, as expected, increase the chances of non-specific amplification.

Through the software Minitab, we can consider all three models—minimize TTR and initial time of amplification (Xt) of positive reactions and minimize Nmax of negative reactions—into account to make the better decision for each variable analyzed. With Minitab, we can aggregate the importance of each response analyzed to give a better fit taking into consideration all the response parameters. Here, the three responses are given the same importance (a weight of 1), however, considering that in two of the three responses analyzed, the parameter time (TTR and initial time) counts as $${\raise0.5ex\hbox{$\scriptstyle 2$} \kern-0.1em/\kern-0.15em \lower0.25ex\hbox{$\scriptstyle 3$}}$$ of the result to choose the variables. Taking all three models into account, the software suggests the optimal levels. The best factors after this round of optimization were onefold primer concentration, 0.5-fold of *Bst* (0.4 U/μL), 8 mM of magnesium sulfate, and 400 mM of betaine, with a temperature reaction of 67 °C. The primer combination chosen was the 1AN (gene N and Orf1ab sequence).

Using the optimized condition mentioned above, we did not observe false-positive results even after a 60-min amplification (Supplementary file).

### Evaluation of different pH indicators on remote diagnostics device

The pH ranges of color shift for each indicator dye tested are shown in Table 1.

**Table 1:** pH indicator dyes tested and their ranges of detection.

pH indicator dye	pH range of detection	Color change
α-Naphtholphthalein	7.3–8.7	Colorless–greenish blue
Bromocresol purple	5.2–6.8	Yellow–pink
Bromothymol blue	6.0–7.6	Yellow–deep blue
Chlorophenol red	4.8–6.7	Yellow–purple
Cresol red	7.2–8.8	Yellow–reddish purple
Metacresol purple	7.4–9.0	Yellow–purple
O-Cresolphthalein	8.2–9.8	Colorless–purple
Phenol red	6.8–8.2	Yellow–pink

We analyzed the pH indicators based on the complexity to prepare stock solutions, stability over time and temperatures, dye precipitation, and pH range against the color shift. We evaluated the dyes in the Molecular Hilab^Ⓡ^ device in various reaction volumes since they can influence the saturation of the color. According to the manufacturer, the *Bst* enzyme is stable in pHs from 5 to 10, dyes that were outside this range or too close to both low and high ends were disregarded as potential indicators. Figure S1 shows the color gradient for each pH indicator dye tested.

Metacresol purple did not dissolve completely in ultrapure type 1 nuclease-free water or TE buffer, so it was discarded from the subsequent tests. Since the Molecular Hilab^Ⓡ^ device displays the reaction result as color time series, using pH indicator dyes that start as a colorless solution could be interpreted as non-insertion of the reaction tubes. Because of that, o-cresolphtalein and α-naphtholphthalein were discarded as potential indicators.

Bromothymol blue and bromocresol purple showed a good color change (from deep blue and purple to yellow, respectively) and stability. Cresol red and chlorophenol red had similar results to phenol red, which is already used in NEB’s LAMP Master mix. After the screening, bromothymol blue was elected as the most stable pH indicator, exhibiting a great color shift, from blue to yellow, which corresponds to a difference of 180° in the hue scale. The optimal concentration of the bromothymol blue in the RT-LAMP reaction was tested and defined at 100 μM—since concentrations below it resulted in light blue color, and above it increased the reaction TTR (data not shown).

### Effect of pH on reaction times

We tested the addition of 1–4 µL of 10 mM KOH (potassium hydroxide) to evaluate the influence of starting pH of the reaction. The parameters analyzed were TTR and Nmax. Interestingly, no significant difference was observed in both parameters. However, the condition with the lowest TTR and the higher Nmax was obtained with 1.2 mM of KOH in the final reaction. The Nmax was approximately 54 (± 4) with a TTR of 420 s when a positive control was used (1 × 10^6^ genome equivalents/reaction) (Fig. S2).

### Combining different dyes to control remote procedures

As a Point-of-Care test, our objective was to develop a kit that would minimize and simplify the manipulation for the operator. Aiming to facilitate the procedure, we tested some combinations of dyes in the sample solution. By doing so, the operator would be able to see if the sample was added to the reaction tube and if the volume pipetted was correct, avoiding false-negative results by a lack of sample addition. Since previous works had shown that a combination of pH indicator dyes could be used in LAMP reactions^8^, we tested combining bromothymol blue with phenol red or cresol red in our reactions.

**Figure 4:**
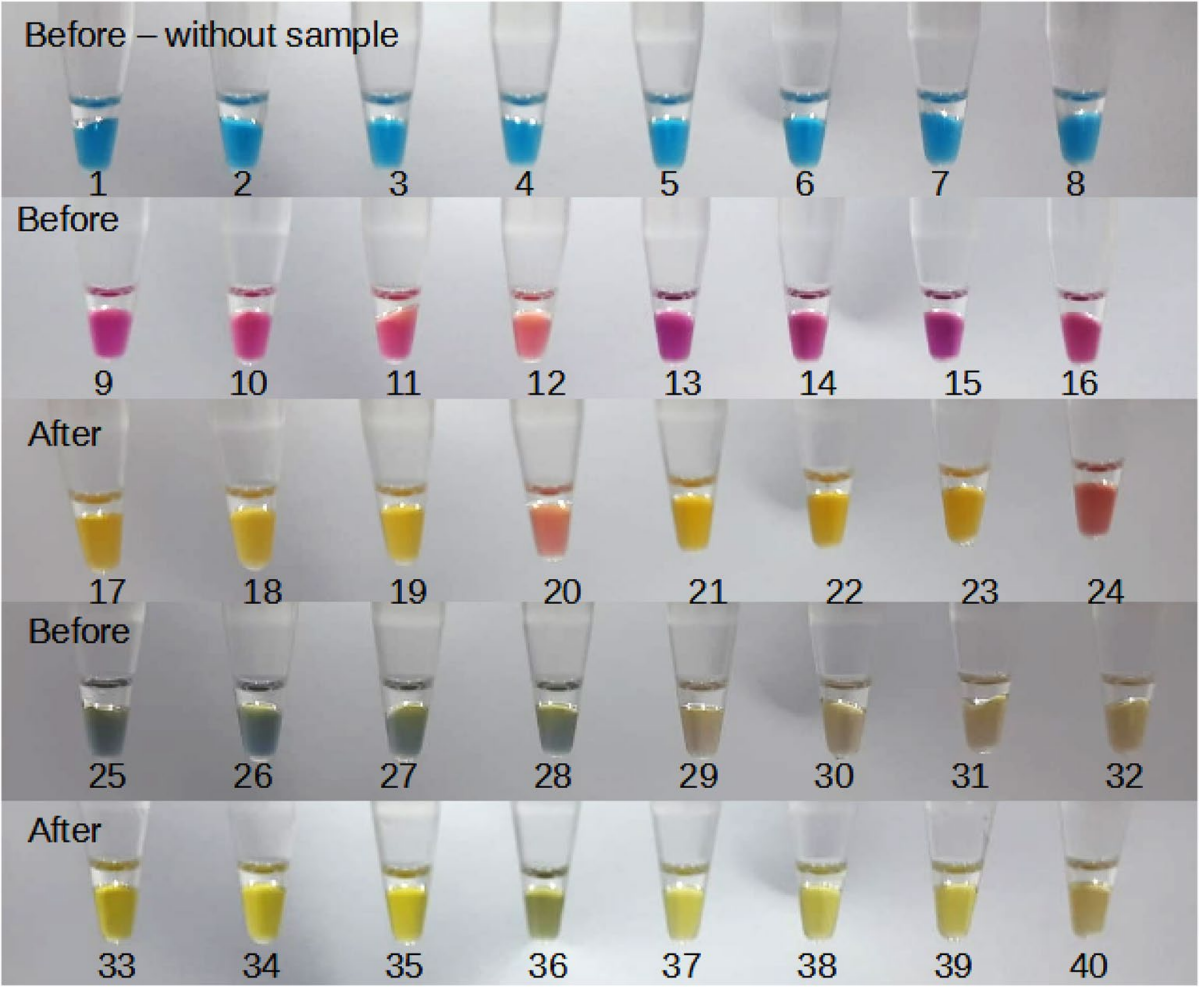
Reactions with different dyes before and after incubation at 67 °C for 30 min. Tubes 1–8 contained only bromothymol blue, showing the color of the reaction before the sample was added. The sample solutions contained either phenol red or cresol red. Tubes 9–16 represent tubes 1–8 after the sample addition with a solution containing phenol red, and tubes 17–24 are after incubation at 67 °C for 30 min. Tubes 25–32 represent tubes 1–8 after the sample addition with a solution containing cresol red, and after an incubation of 67 °C for 30 min, the results were the tubes 33–40. Tubes 12, 16, 28, and 32 were non-template controls. The final concentration of dyes in the tubes was: tubes 1–4 contained 50 μM bromothymol blue; 5–8 contained 100 μM bromothymol blue; 9–12 and 17–20 contained 50 μM bromothymol blue and 100 μM phenol red; 13–16 and 21–24 contained 100 μM bromothymol blue and 100 μM phenol red; 25–28 and 33–36 contained 100 μM bromothymol blue and 100 μM cresol red; 29–32 and 37–40 contained 50 μM bromothymol blue and 100 μM cresol red.

The combination of bromothymol blue and cresol red resulted in a muddy blueish color after sample addition (Fig. 4—tubes 25–32). After a 30-min amplification, the color in positive control tubes (33–35 and 37–39) could be slightly distinguished from the non-template controls (NTC) (tubes 36 and 40). Phenol red was elected as the best dye to be used in the composition of the sample solution. After the sample was added, the color of the reaction went from blue (tubes 1–8) to purple (tubes 9–16), being a perceptible color change to the operator. In the case of amplification the final color was a bright yellow (tubes 17–19; 21–23) and was clearly distinguished from NTC tubes (20 and 24) (Fig. 4). Besides, we were able to obtain a satisfactory Nmax and TTR (< 600 s) with the combination of the dyes.

We did not observe any false-positive reaction in a 30-min amplification.

### Effect of the initial pH of sample solution

Considering that the colorimetric LAMP reaction used in this paper is pH dependent, we ought to analyze whether the pH of the sample solution could interfere with the reaction parameters like Nmax and TTR. Sample solutions were tested in five different pHs ranging from 7 to 9, with a 0.5 increase. Without any adjustment, the solution had a pH of approximately 8.5, so we had to add either NaOH or HCl to achieve the desired pH. After swirling a nasopharyngeal swab sample into the solution, we verified that the pink color intensified, indicating some basification.

All reactions became positive when testing with internal control primers for human rActin (Table S2), however, a higher pH of the sample solution caused an increase in the TTR, as was expected (Table S3). Sample solutions with a pH of 7.0–7.5 showed higher consistency between replicates and lower TTR values (642 ± 54 and 714 ± 24 s, respectively).

### Effect of Tris concentration in the LAMP reaction

Colorimetric reactions that rely on pH change are not compatible with buffer solutions, and usually contain only carryover from enzyme storage buffers. However, we’ve noticed that when using highly concentrated enzymes (stock solutions at 120 U/μL), thus having a lower buffer carryover, reactions containing bromothymol blue quickly acidified. About 15 min in ice was enough to cause a visible change in the reaction color from blue to greenish. We tested whether there was an optimal Tris concentration that would prevent color change during the preparation of the tubes without interfering with the amplification step. We analyzed the delta value (Nmax) and the initial time of the amplification (Xt) obtained in each condition right after the production of the reaction mix.

It was possible to observe that with increasing Tris concentration the initial time of amplification increased as well (Fig. S3). Due to the buffer characteristic of Tris between pH range from 6 to 8, the pH of the reactions, and consequently the color of it, were more difficult to change as the concentration of Tris was higher. The Tris concentration and the Nmax were inversely proportional, a higher quantity of Tris resulted in a lower delta (Nmax) of the reaction color when compared to the lowest concentration of Tris tested (510 μM).

### Effect of mineral oil on reactions without lid heating

**Figure 5:**
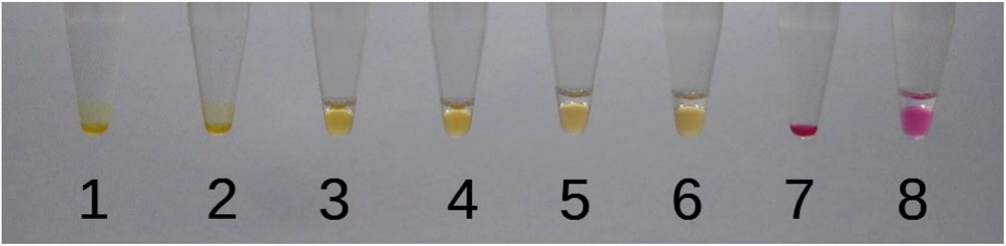
Effect of the mineral oil after amplification at 67 °C for 60 min. In tubes 1, 2, and 7 there was no addition of the mineral oil; tubes 3 and 4 received 5 μL of mineral oil, and tubes 5, 6, and 8 received 10 μL of mineral oil. Tubes 1–6 received SARS-CoV-2 positive control and tubes 7 and 8 were negative controls.

There was no significant difference with the different methods of adding the sample, i.e., the presence of the mineral oil was not an issue when adding the sample to the reaction. Furthermore, in the conditions where 0 or 5 μL of mineral oil were added to the reaction, evaporation still occurred, as small droplets could be observed in the microtube cap and the volume in the bottom of the microtube decreased (Fig. 5). We observed that without the mineral oil it wasn’t possible to achieve a stabilization of the Nmax since the evaporation occurred during the amplification, increasing the saturation of the captured color, leading to a higher Nmax.

The final protocol was set at the addition of 12 μL of mineral oil to a 20 μL reaction, as no droplets were observed in the microtube cap—the volume in the bottom of the tube stayed the same during the whole test—and it was possible to achieve a stabilization of the Nmax value. We did not observe a significant difference in TTR or sensitivity when mineral oil was added to the reaction.

### Limit of detection (LoD)

**Figure 6:**
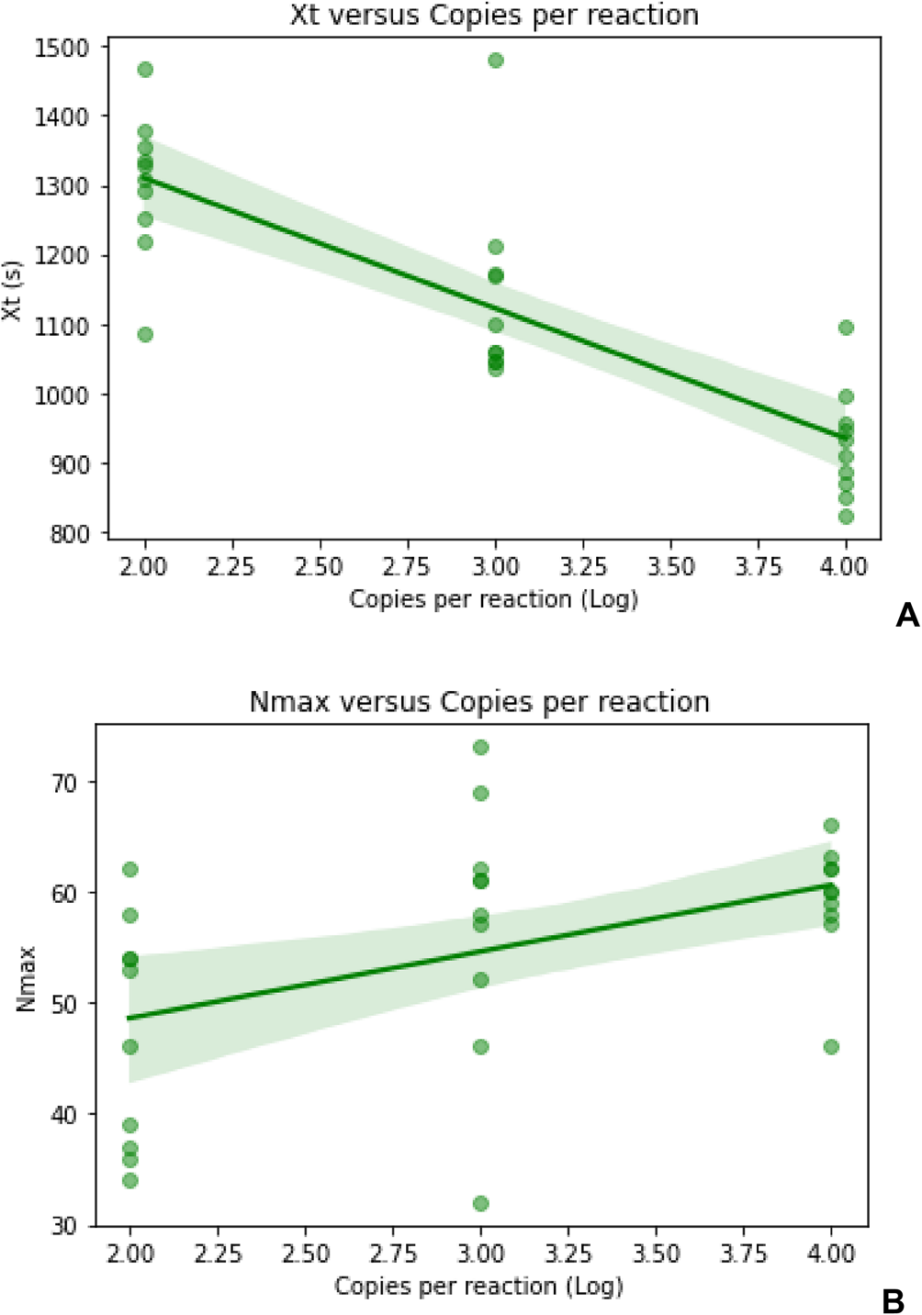
Limit of detection of the test using a control solution containing plasmids pUC57 cloned with the synthetic DNA sequences corresponding either to the sites of gene N or Orf1ab. (**A**) In the x-axis is the dilution of the control solution in copies per reaction in Log and the y-axis is the Xt (initial time of amplification in seconds) of the reactions. (**B**) In the x-axis is the dilution of the control solution in copies per reaction and the y-axis is the Nmax of the reaction.

The limit was established at 5 genome equivalents per μL with 100% of positivity (10/10) with a Nmax mean of 54 (± 26) and the initial time of amplification (Xt) of approximately 1302 s (± 396) (Fig. 6 and Fig. S4). More experiments simulating transportation and storage are being executed to analyze if the test sensitivity remains the same.

### Summary of the optimization

In Table 2 we summarize the optimized composition of the reaction buffer and sample solution. Nasopharyngeal swab samples can be heat inactivated at 95 °C for 10 min or added to the reaction without any treatment. After sample addition, the amplification should be done at 67 °C for 30 min.

**Table 2:** Summary of sample solution and reaction buffer compositions.

Sample solution (500 μL; pH 7.0–7.5)	
Phenol red	500 µM
TE buffer pH 8.0	10% (v/v)
Tween-20	0.1% (v/v)
KOH	Enough to reach pH 7.0–7.5
Ultrapure type I nuclease-free water	q.s. 500 μL

Reaction buffer (20 μL)	
FIP	1.6 µM
BIP	1.6 µM
F3	0.2 µM
B3	0.2 µM
LF	0.4 µM
LB	0.4 µM
dNTP	1.4 mM
*Bst* 2.0 WarmStart® DNA polymerase	0.4 U/µL
WarmStart® RTx Reverse Transcriptase	0.3 U/µL
Betaine	400 mM
Guanidine hydrochloride pH 8.0	40 mM
KCl	50 mM
MgSO_4_	8 mM
Tween-20	0.1% (v/v)
Bromothymol blue	100 µM
KOH	1.2 mM
Sample	4 µL
Ultrapure type I nuclease-free water	q.s. 20 μL
Mineral oil	12 µL

## Discussion

Colorimetric RT-LAMP reactions have revolutionized molecular POCT testing, enabling cheap instrumentation with high sensitivity and specificity—characteristic of molecular assays like qPCR^9^. Although commonly used because of its easy differentiation between negative and positive reactions by naked-eye observation, colorimetric reactions also allow the substitution of expensive and unstable fluorophores and filter setups for simpler data acquisition systems. In our study, we’ve used the Hilab® Molecular device, which features an RGB detection coupled with white LEDs and internet connectivity through WiFi or USB, providing simultaneous monitoring of the reaction and a remote evaluation of the assay by health professionals. In the present study, we have developed a novel formulation optimized for remote POCT tests analysis, using the Hilab® Molecular device.

The quantification of amplified genetic material by qPCR reactions can be measured by temperature cycles, where the amount of DNA is doubled each cycle, making it easier to interpret reaction progress and estipulate the initial sample load. On the other hand, RT-LAMP reactions occur at a single temperature and have complex kinetics when compared to qPCR. However, colorimetric LAMP reactions can be described as parameters like time to reaction (TTR), initial time of amplification (Xt), and maximum color change (Nmax). Nonetheless, we have shown that these parameters are still helpful for the empirical modeling of a quantitative measurement of amplification rate using DoE. Acceptable conditions were obtained for five continuous and two categorical variables, with about 200 unreplicated test conditions. Even with outliers removal, the obtained models were acceptable and were successfully used to obtain optimized conditions for our specific assay conditions.

Among all the parameters tested, we observed that the primer sequence—aiming the N, ORF1ab, or E gene—, *Bst*, primer, and magnesium sulfate concentrations were the most significant (α = 0.05). This is consistent with other papers that tested component optimization, which also saw that increasing the enzyme and MgSO_4_ concentration^10,11^ improved the performance of the reaction. In our case, the best concentration was 0.4 U/μL of *Bst* and 8 mM of MgSO_4_. The NEB’s WarmStart master mix for SARS-CoV-2 (which contains the *Bst* DNA polymerase 2.0) recommends a range of Tris from 0.5 and 5 mM^12^, we found that the lowest concentration of Tris that could be achieved (510 μM) gives a lower TTR. This is in agreement with the buffer characteristics of Tris since the reaction has to be weakly buffered to permit the color change of the pH-dependent dye. They also recommend that the pH of the master mix should be between 7.5 and 9.0. We tested the KOH concentration to define an initial pH of the reaction that gives a lower TTR and a higher Nmax. We saw that the KOH concentrations tested had no statistically significant difference in the TTR or Nmax, although a final concentration of 1.2 mM gave a slightly better color shift (Nmax). We also addressed the pH adjustment of the sample solution by measuring the TTR and Nmax of the reaction right after its preparation and by the decrease of its pH over time. A sample solution with a pH between 7 and 7.5 shows a lower TTR of the reaction and has a better stabilization of the pH during storage. Furthermore, higher pH demonstrated a significant pH reduction through a few weeks of storage (data not shown).

Guanidine hydrochloride pH 8.0 has been shown to improve the LAMP reaction^13^. We observed that the adjustment of the Gu-HCl pH to 8.0 didn’t significantly impact the TTR or Nmax of the reaction. Additionally, the Gu-HCl pH decreased over time, which is incompatible with long time storage, and an adjustment just before use is incongruous with a POC test. Another additive tested was betaine which has been used in PCR and LAMP techniques to improve the specificity of the assay^14,15^. Using a concentration of 400 mM betaine we did not see unspecific amplification, differently than seen in another study^15^.

A combination of dyes in the reaction mix has been previously tested to expand the application of colorimetric LAMP^8^, however, here we use the combination of different dyes as control of pipetting to guarantee that the operator has added the sample to the reaction tube, preventing false negatives by lack of sample addition. The sample solution contains phenol red and after the addition of the sample into the reaction mix—which contains bromothymol blue—the color turns purple. Quality control of remotely performed tests is of utmost importance, especially in the case of molecular diagnosis. The inclusion of the dye in the sample buffer does not appear to affect the efficiency of sample treatment and is unlikely to affect heat inactivation. Among the eight dyes tested, we chose the bromothymol blue dye to compose the reaction mix and the phenol red dye for the sample solution. The inverse (phenol red in the reaction mix and bromothymol blue in the sample solution) was not approved because the sample solution would turn darker and unattractive as a diagnostics product. Both dyes were stable, with a pH range compatible with our reaction and the color change was better detected in the RGB system. Besides, these two dyes have been widely used in colorimetric LAMP reactions^4,8,16,17^.

The Hilab® Molecular device does not feature a heated lid to facilitate production and increase robustness by reducing the number of moving parts. This, however, makes reactions susceptible to excessive evaporation, leading to possible misinterpretation of colorimetric reactions caused by the increase of color saturation. To mitigate this problem, mineral oil for PCR was tested to prevent evaporation^18^. We’ve found that the addition of the oil can completely inhibit evaporation and stabilizing color, with no detrimental effects to signal or time to reaction. The presence of mineral oil has not affected the process of sample transfer, whether it was added on top or under the mineral oil layer. Homogenizing the solution with a pipette, or by knocking on the sides of the tube, also did not affect the reaction efficiency. The oil layer was re-established after a few seconds. The mineral oil can be added beforehand and the reaction can be frozen with the oil. Besides that, the oil allows for the reaction to be run in simpler settings, e.g. in a dry block.

The limit of detection (LoD) achieved was 5 copies per μL with 100% positivity using synthetic control—similar to other RT-LAMP assay^19^. Previous experiments done in our laboratory using the NEB’s WarmStart Colorimetric LAMP 2X Master Mix with primers targeting genes N and E of SARS-CoV-2 could not achieve a limit of detection below 50 copies per μL (data not shown). The limit of detection of RT-PCR tests using purified samples varies between 0.08^19^ and 14^20^ copies per μL^21–23^. Comparing our LoD with other molecular tests using a direct swab, our limit of detection was 25,000 copies per mL when the LoD of the commercial tests were 60,000–540,000 NDU per mL^24^—viral RNA copies/mL is equivalent to genome copy equivalents/mL (GCE/mL) or nucleic acid detectable units/mL (NDU/mL)^25^. The results obtained were not compared with RT-qPCR assay since our technique uses unpurified samples.

Tests of stability are yet to be executed to verify if the LoD remains the same after a long period of storage. Clinical validation of the test using patient samples positive for COVID-19 is also required.

It is important to be mindful that the results found here were achieved using 4 μL of sample solution to a final reaction volume of 20 μL. The results may be different if the proportion of sample added or the sample solution composition changes. Besides that, although increasing the total volume of the reaction can improve the sensitivity^26^, this may increase the cost of the test.

## Conclusion

Here we’ve successfully optimized reaction conditions for colorimetric detection of nucleic acids in POCT settings, with tenfold higher sensitivity compared to the WarmStart Colorimetric LAMP Master mix from NEB. Our master mix recipe can maximize color signal differences, minimize the amplification time of target sequences, control the sample addition, and may be used in a refrigerated setting. It is also able to accommodate longer reaction times without evaporation of the volume, even without the use of a heated lid, thanks to the addition of mineral oil. Non-specific amplification was also mitigated by Response Surface Methodology (RSM) optimization. These enhancements will allow for more cost-effective screening tests in a wide range of possible configurations.

## Materials and methods

### Reagents and equipment

*Bst* 2.0 WarmStart® DNA Polymerase (M0538), Antarctic Thermolabile UDG (M0372), WarmStart® RTx Reverse Transcriptase (M0380), as well as dNTPs (N0446S and N0459S) were acquired from New England Biolabs (NEB). Phenol Red (114529), Mineral Oil (M5904), Potassium Chloride (P9541), Potassium Hydroxide (P5958), Magnesium Sulfate (M3409), Tween-20 (P9416), Guanidine Hydrochloride (G3272), Tris-Hydrochloride (93363) and Betaine (B0300) were acquired from Sigma-Aldrich. Ultrapure Water type I (10977) and TE buffer pH 8.0 (93283) were acquired from Invitrogen. Bromothymol Blue (AB08416RA), Cresol Red (VC09256RA), Chlorophenol Red (VC06226RA), Bromocresol Purple (PB06593RA), Metacresol Purple (PM06383RA), α-Naphtholphthalein (AN07911RA), and O-Cresolphthalein (C05368RA) dyes were acquired from Êxodo Científica.

### Primers and positive controls

The primer sequences used targeted the N gene, the E genes^13^, or the Orf1ab sequence^27^ from SARS-CoV-2. A set of primers targeting the human ACTB mRNA sequence were used as the internal control (Table S2). Primers were ordered from Exxtend with HPLC purification and resuspended to 200 μM in ultra-pure type 1 nuclease-free water. To facilitate pipetting, primers were prepared as a tenfold concentrate primer mix. The internal control primer mix was constituted only by ACTB primers, while the SARS-CoV-2 primer mix was composed of a combination of two sets of primers targeting either N and E genes, N and Orf1ab genes, or E and Orf1ab genes. Each mix had 16 µM FIP and BIP primers, 2 μM F3 and B3 primers, and 4 μM loop primers for each target used.

Lyophilized positive controls were obtained from GenScript and consisted of pUC57 plasmids cloned in the EcoRV site with either gene N, gene E, or Orf1ab sequence from SARS-CoV-2 (Table S2). Positive controls were diluted with ultra-pure type 1 nuclease-free water to an intermediate concentration and then mixed with sample solution (described below) to achieve a final concentration of 10^6^ copies/μL of each target.

### Samples solution and nasopharyngeal swabs

Sample solution, unless stated otherwise, consisted of 10% TE Buffer pH 8.0 (10 mM Tris and 1 mM EDTA), 0.1% Tween-20, and 500 μM Phenol Red. The sample solution was added to the reaction in a volume of 4 μL to obtain a final concentration of 100 μM of the colorimetric dye.

In experiments where we used primers targeting ACTB mRNA, nasopharyngeal (NP) swab samples of asymptomatic volunteers were collected by trained personnel. Samples collected were then swirled in a 1.5 mL microcentrifuge tube containing 500 μL of sample solution, and thermally inactivated at 95 °C for 10 min. All samples used in this study were obtained under the signature of free and clarified consent terms.

### RT-LAMP reactions

RT-LAMP reactions were carried out, unless stated otherwise, with a final concentration of 1.6 µM FIP and BIP primers, 0.2 μM F3 and B3 primers, and 0.4 μM loop primers of each target gene. Also present were 1.4 mM dNTP (1.4 mM dATP, dCTP, dGTP and 0.7 mM dTTP, dUTP), 0.4 U/μL of *Bst* 2.0 WarmStart® DNA Polymerase, 0.3 U/μL of WarmStart® RTx Reverse Transcriptase, 0.005 U/μL Antarctic Thermolabile UDG, 400 mM Betaine, 40 mM Guanidine Hydrochloride pH 8.0, 10 mM KCl, 8 mM MgSO_4_, 0.1% (v/v) Tween-20, 100 μM of Bromothymol Blue, and 4 μL of the sample solution, with a total reaction of 20 μL.

The reaction protocol tested was set at 67 °C for 30 min, unless stated otherwise. Since the reaction has two pH indicators included—bromothymol blue and phenol red—the readout of a positive or negative result was taken by the color change in the tubes. In the case of a positive reaction, the initial color changed from purple to yellow by the decrease of the reaction’s pH, caused by the release of protons as dNTPs were incorporated into the newly synthesized DNA strand.

### Design of experiments analysis

To determine optimal parameters for our custom RT-LAMP formulation, a Design of Experiments approach was taken. We chose a Response Surface Methodology (RSM), to fine-tune the value of five continuous factors (DNA polymerase, magnesium sulfate, primers, and betaine concentrations, as well as reaction temperature) and two categorical factors (pH adjusting of guanidine hydrochloride [yes/no], and primer sequence combination [N/E, N/Orf1ab or E/Orf1ab]). Betaine and magnesium sulfate high and low levels were modeled as millimolar concentrations, while primer concentrations were modeled as fractions from the standard recipe. Polymerase concentrations were modeled as fractions from a maximum concentration of 0.8 U/μL. Temperature reaction high and low levels were set as + 2° or − 2° from the original 65 °C temperature.

Minitab 19 was used to generate the experimental matrix, randomize run order and analyze data. For each condition, we used a positive (1 × 10^6^ genome equivalents/reaction) and a negative control reaction to measure reaction efficiency and non-specific amplification rates for each condition. The response factors analyzed were Time To Reaction (TTR), Nmax, and initial time of amplification (Xt) for both positive and negative reactions.

### Effect of pH indicator dyes and initial pH of the reaction

Based on the published literature^28,29^ we tested other pH indicator dyes such as bromothymol blue, phenol red, cresol red, chlorophenol red, bromocresol purple, metacresol purple, α-naphtholphthalein, and O-cresolphthalein in final concentrations ranging from 50 to 200 μM. Stock solutions were prepared as 2 mM of the dye in 1 mM TE buffer and diluted to the desired concentration with ultrapure type I nuclease-free water. pHs gradients were obtained by mixing the colorimetric dyes with either 1 M NaOH or HCl.

Given that our RT-LAMP test is pH-dependent, we hypothesized that the initial pH of the solution could affect the TTR or the Nmax of the reaction. To verify this, we tested the optimal initial pH of the reaction, adjusting it with 10 mM KOH to a final concentration of 0.4–1.6 mM KOH. Tubes contained primers targeting genes N and Orf1ab and positive controls as described above.

### Combining different dyes to control remote procedures

We tested a combination of bromothymol blue with cresol red and phenol red in final concentrations ranging from 50 to 200 μM, in both sample and reaction solutions. A nasopharyngeal swab collected was used as a sample, and reactions had primers targeting ACTB mRNA. Non-template controls were constituted only by sample solution. The dye was selected based on the color change after adding the sample to the LAMP reaction and at the end of the positive reaction.

### Effect of the initial pH of sample solution

We prepared sample solutions in pHs ranging from 7 to 9 with a 0.5 increase between conditions. Each pH condition had 1 tube as a negative control and a technical triplicate with nasopharyngeal swab samples. Tubes contained primers targeting ACTB mRNA.

### Effect of Tris concentration in the LAMP reaction

Since our protocol was based on the change of the pH reaction, we tested the ideal concentration of Tris in the final reaction. The enzymes used in the LAMP reaction and the reagents of the sample solution contained Tris in their compositions, so the minimal concentration of Tris that could be achieved in the reaction was 510 μM—the lower concentration tested—and the highest being 1000 μM (NEB claims it’s Warmstart® RT-LAMP master mix works with up to 1000 μM of Tris). Different volumes of 10 mM Tris buffer were added to each reaction to achieve the final concentrations tested. Tubes contained primers targeting genes N and Orf1ab and a diluted positive control (1000 genomic copies/reaction) was used to verify the effect of Tris buffer while simulating low viral load samples.

### Effect of mineral oil on reactions without lid heating

As the Molecular Hilab® system does not feature a heated lid, evaporation of the solution happens, which may affect reaction efficiency and color data acquisition. To avoid this problem, we tested the addition of 0–15 μL per reaction of mineral oil. We also tested whether the presence of the mineral oil could somehow hinder the addition of the sample in the reaction mixture, by adding the sample through, above, and at the bottom of the oil, intentionally making bubbles in the tube. Tubes contained primers targeting genes N and Orf1ab and positive controls were used as previously described.

### Limit of detection (LoD)

To verify the limit of detection of the RT-LAMP reaction we used positive controls in concentrations ranging from 10 to 10,000 genome equivalents per reaction. Each dilution point was performed in 10 technical replicates. The controls were diluted in the sample solution and the reactions were carried out at 67 °C for 30 min in the Hilab® Molecular device as previously described. We established the LoD as the maximum dilution where it was possible to obtain 100% of positivity, i.e., amplification of the targets.

### Quantitative analysis

To quantify the results of each optimized condition internally developed software was used. The software was able to recognize a table sheet of hexadecimal colors and build a graph based on the color change of the reaction. After the graph was built, the operator could manually set the initial time of the amplification (Xt), so the software would do a background removal and build a sigmoid curve using a 4-parameter polynomial regression of the data. The software also returned three parameters for each tube: Nmax, which is the delta value of the amplification curve, TTR, which is the time to achieve 50% of the amplification delta, and R, which is related to the efficiency of the reaction and represents how fast the reaction reaches the plateau. We also analyzed the initial time of amplification (Xt) in some cases. These parameters were calculated for every test reported here and were used to decide which condition would be the best for each experiment.

### Remote molecular diagnostic device

The Molecular Hilab® system was used to monitor colorimetric reactions remotely. The device is composed of a thermoblock and an RGB sensor, being able to heat inactivate and cool the samples, keep the reaction at optimal temperature, and detect the insertion of the reaction tubes. Insertion of the tubes in the device as well as sample handling is done manually by the operator. Reaction data is sent as a color time series. After the collection of the data, two images are generated: an image with the gradual change of the reaction color until the end of the reaction, and a graph built from the color change series, with the curves of each tube over time. With the device, it is possible to analyze the color of the reaction tubes with more precision and robustness than by the naked eye, for example. These images along with the anamnesis questionnaire are taken into consideration to give the final result of the test by biomedical professionals within 1 min.

## Supplementary Information

Supplementary Information 1.

Supplementary Information 2.

## Data Availability

The data that support the findings of this study are available from the corresponding author, Raddatz, W. B., upon reasonable request.
