# Supplementary material for: Development of an optimized colorimetric RT-LAMP for SARS-CoV-2 assay with enhanced procedure controls for remote diagnostics
Source: Sci Rep. 2022 Dec 11;12:21424. doi: 10.1038/s41598-022-25872-1 (PMC9741705; doi:10.1038/s41598-022-25872-1)
Supplement: Supplementary file 1 — Supplementary Information 1. [file 41598_2022_25872_MOESM1_ESM.docx]

1. **SUPP INFO**

**Table S1** - Experimental matrix of the Response Surface Methodology experiment.

| **Primer (X)** | **Bst (U/μL)** | **MgSO4 (mM)** | **Betaine (mM)** | **Temperature (°C)** | **Primer (type)** | **Gua-HCl (with or without pH adjustment)** |
| --- | --- | --- | --- | --- | --- | --- |
| 0.4 | 0.4 | 7 | 100 | 66 | 1AN | Yes |
| 0.8 | 0.4 | 7 | 100 | 64 | 1AN | Yes |
| 0.4 | 0.8 | 7 | 100 | 64 | 1AN | Yes |
| 0.8 | 0.8 | 7 | 100 | 66 | 1AN | Yes |
| 0.4 | 0.4 | 9 | 100 | 64 | 1AN | Yes |
| 0.8 | 0.4 | 9 | 100 | 66 | 1AN | Yes |
| 0.4 | 0.8 | 9 | 100 | 66 | 1AN | Yes |
| 0.8 | 0.8 | 9 | 100 | 64 | 1AN | Yes |
| 0.4 | 0.4 | 7 | 300 | 64 | 1AN | Yes |
| 0.8 | 0.4 | 7 | 300 | 66 | 1AN | Yes |
| 0.4 | 0.8 | 7 | 300 | 66 | 1AN | Yes |
| 0.8 | 0.8 | 7 | 300 | 64 | 1AN | Yes |
| 0.4 | 0.4 | 9 | 300 | 66 | 1AN | Yes |
| 0.8 | 0.4 | 9 | 300 | 64 | 1AN | Yes |
| 0.4 | 0.8 | 9 | 300 | 64 | 1AN | Yes |
| 0.8 | 0.8 | 9 | 300 | 66 | 1AN | Yes |
| 0.2 | 0.6 | 8 | 200 | 65 | 1AN | Yes |
| 1 | 0.6 | 8 | 200 | 65 | 1AN | Yes |
| 0.6 | 0.2 | 8 | 200 | 65 | 1AN | Yes |
| 0.6 | 1 | 8 | 200 | 65 | 1AN | Yes |
| 0.6 | 0.6 | 6 | 200 | 65 | 1AN | Yes |
| 0.6 | 0.6 | 10 | 200 | 65 | 1AN | Yes |
| 0.6 | 0.6 | 8 | 0 | 65 | 1AN | Yes |
| 0.6 | 0.6 | 8 | 400 | 65 | 1AN | Yes |
| 0.6 | 0.6 | 8 | 200 | 63 | 1AN | Yes |
| 0.6 | 0.6 | 8 | 200 | 67 | 1AN | Yes |
| 0.6 | 0.6 | 8 | 200 | 65 | 1AN | Yes |
| 0.6 | 0.6 | 8 | 200 | 65 | 1AN | Yes |
| 0.6 | 0.6 | 8 | 200 | 65 | 1AN | Yes |
| 0.6 | 0.6 | 8 | 200 | 65 | 1AN | Yes |
| 0.6 | 0.6 | 8 | 200 | 65 | 1AN | Yes |
| 0.6 | 0.6 | 8 | 200 | 65 | 1AN | Yes |
| 0.4 | 0.4 | 7 | 100 | 66 | 1AE | Yes |
| 0.8 | 0.4 | 7 | 100 | 64 | 1AE | Yes |
| 0.4 | 0.8 | 7 | 100 | 64 | 1AE | Yes |
| 0.8 | 0.8 | 7 | 100 | 66 | 1AE | Yes |
| 0.4 | 0.4 | 9 | 100 | 64 | 1AE | Yes |
| 0.8 | 0.4 | 9 | 100 | 66 | 1AE | Yes |
| 0.4 | 0.8 | 9 | 100 | 66 | 1AE | Yes |
| 0.8 | 0.8 | 9 | 100 | 64 | 1AE | Yes |
| 0.4 | 0.4 | 7 | 300 | 64 | 1AE | Yes |
| 0.8 | 0.4 | 7 | 300 | 66 | 1AE | Yes |
| 0.4 | 0.8 | 7 | 300 | 66 | 1AE | Yes |
| 0.8 | 0.8 | 7 | 300 | 64 | 1AE | Yes |
| 0.4 | 0.4 | 9 | 300 | 66 | 1AE | Yes |
| 0.8 | 0.4 | 9 | 300 | 64 | 1AE | Yes |
| 0.4 | 0.8 | 9 | 300 | 64 | 1AE | Yes |
| 0.8 | 0.8 | 9 | 300 | 66 | 1AE | Yes |
| 0.2 | 0.6 | 8 | 200 | 65 | 1AE | Yes |
| 1 | 0.6 | 8 | 200 | 65 | 1AE | Yes |
| 0.6 | 0.2 | 8 | 200 | 65 | 1AE | Yes |
| 0.6 | 1 | 8 | 200 | 65 | 1AE | Yes |
| 0.6 | 0.6 | 6 | 200 | 65 | 1AE | Yes |
| 0.6 | 0.6 | 10 | 200 | 65 | 1AE | Yes |
| 0.6 | 0.6 | 8 | 0 | 65 | 1AE | Yes |
| 0.6 | 0.6 | 8 | 400 | 65 | 1AE | Yes |
| 0.6 | 0.6 | 8 | 200 | 63 | 1AE | Yes |
| 0.6 | 0.6 | 8 | 200 | 67 | 1AE | Yes |
| 0.6 | 0.6 | 8 | 200 | 65 | 1AE | Yes |
| 0.6 | 0.6 | 8 | 200 | 65 | 1AE | Yes |
| 0.6 | 0.6 | 8 | 200 | 65 | 1AE | Yes |
| 0.6 | 0.6 | 8 | 200 | 65 | 1AE | Yes |
| 0.6 | 0.6 | 8 | 200 | 65 | 1AE | Yes |
| 0.6 | 0.6 | 8 | 200 | 65 | 1AE | Yes |
| 0.4 | 0.4 | 7 | 100 | 66 | NE | Yes |
| 0.8 | 0.4 | 7 | 100 | 64 | NE | Yes |
| 0.4 | 0.8 | 7 | 100 | 64 | NE | Yes |
| 0.8 | 0.8 | 7 | 100 | 66 | NE | Yes |
| 0.4 | 0.4 | 9 | 100 | 64 | NE | Yes |
| 0.8 | 0.4 | 9 | 100 | 66 | NE | Yes |
| 0.4 | 0.8 | 9 | 100 | 66 | NE | Yes |
| 0.8 | 0.8 | 9 | 100 | 64 | NE | Yes |
| 0.4 | 0.4 | 7 | 300 | 64 | NE | Yes |
| 0.8 | 0.4 | 7 | 300 | 66 | NE | Yes |
| 0.4 | 0.8 | 7 | 300 | 66 | NE | Yes |
| 0.8 | 0.8 | 7 | 300 | 64 | NE | Yes |
| 0.4 | 0.4 | 9 | 300 | 66 | NE | Yes |
| 0.8 | 0.4 | 9 | 300 | 64 | NE | Yes |
| 0.4 | 0.8 | 9 | 300 | 64 | NE | Yes |
| 0.8 | 0.8 | 9 | 300 | 66 | NE | Yes |
| 0.2 | 0.6 | 8 | 200 | 65 | NE | Yes |
| 1 | 0.6 | 8 | 200 | 65 | NE | Yes |
| 0.6 | 0.2 | 8 | 200 | 65 | NE | Yes |
| 0.6 | 1 | 8 | 200 | 65 | NE | Yes |
| 0.6 | 0.6 | 6 | 200 | 65 | NE | Yes |
| 0.6 | 0.6 | 10 | 200 | 65 | NE | Yes |
| 0.6 | 0.6 | 8 | 0 | 65 | NE | Yes |
| 0.6 | 0.6 | 8 | 400 | 65 | NE | Yes |
| 0.6 | 0.6 | 8 | 200 | 63 | NE | Yes |
| 0.6 | 0.6 | 8 | 200 | 67 | NE | Yes |
| 0.6 | 0.6 | 8 | 200 | 65 | NE | Yes |
| 0.6 | 0.6 | 8 | 200 | 65 | NE | Yes |
| 0.6 | 0.6 | 8 | 200 | 65 | NE | Yes |
| 0.6 | 0.6 | 8 | 200 | 65 | NE | Yes |
| 0.6 | 0.6 | 8 | 200 | 65 | NE | Yes |
| 0.6 | 0.6 | 8 | 200 | 65 | NE | Yes |
| 0.4 | 0.4 | 7 | 100 | 66 | 1AN | No |
| 0.8 | 0.4 | 7 | 100 | 64 | 1AN | No |
| 0.4 | 0.8 | 7 | 100 | 64 | 1AN | No |
| 0.8 | 0.8 | 7 | 100 | 66 | 1AN | No |
| 0.4 | 0.4 | 9 | 100 | 64 | 1AN | No |
| 0.8 | 0.4 | 9 | 100 | 66 | 1AN | No |
| 0.4 | 0.8 | 9 | 100 | 66 | 1AN | No |
| 0.8 | 0.8 | 9 | 100 | 64 | 1AN | No |
| 0.4 | 0.4 | 7 | 300 | 64 | 1AN | No |
| 0.8 | 0.4 | 7 | 300 | 66 | 1AN | No |
| 0.4 | 0.8 | 7 | 300 | 66 | 1AN | No |
| 0.8 | 0.8 | 7 | 300 | 64 | 1AN | No |
| 0.4 | 0.4 | 9 | 300 | 66 | 1AN | No |
| 0.8 | 0.4 | 9 | 300 | 64 | 1AN | No |
| 0.4 | 0.8 | 9 | 300 | 64 | 1AN | No |
| 0.8 | 0.8 | 9 | 300 | 66 | 1AN | No |
| 0.2 | 0.6 | 8 | 200 | 65 | 1AN | No |
| 1 | 0.6 | 8 | 200 | 65 | 1AN | No |
| 0.6 | 0.2 | 8 | 200 | 65 | 1AN | No |
| 0.6 | 1 | 8 | 200 | 65 | 1AN | No |
| 0.6 | 0.6 | 6 | 200 | 65 | 1AN | No |
| 0.6 | 0.6 | 10 | 200 | 65 | 1AN | No |
| 0.6 | 0.6 | 8 | 0 | 65 | 1AN | No |
| 0.6 | 0.6 | 8 | 400 | 65 | 1AN | No |
| 0.6 | 0.6 | 8 | 200 | 63 | 1AN | No |
| 0.6 | 0.6 | 8 | 200 | 67 | 1AN | No |
| 0.6 | 0.6 | 8 | 200 | 65 | 1AN | No |
| 0.6 | 0.6 | 8 | 200 | 65 | 1AN | No |
| 0.6 | 0.6 | 8 | 200 | 65 | 1AN | No |
| 0.6 | 0.6 | 8 | 200 | 65 | 1AN | No |
| 0.6 | 0.6 | 8 | 200 | 65 | 1AN | No |
| 0.6 | 0.6 | 8 | 200 | 65 | 1AN | No |
| 0.4 | 0.4 | 7 | 100 | 66 | 1AE | No |
| 0.8 | 0.4 | 7 | 100 | 64 | 1AE | No |
| 0.4 | 0.8 | 7 | 100 | 64 | 1AE | No |
| 0.8 | 0.8 | 7 | 100 | 66 | 1AE | No |
| 0.4 | 0.4 | 9 | 100 | 64 | 1AE | No |
| 0.8 | 0.4 | 9 | 100 | 66 | 1AE | No |
| 0.4 | 0.8 | 9 | 100 | 66 | 1AE | No |
| 0.8 | 0.8 | 9 | 100 | 64 | 1AE | No |
| 0.4 | 0.4 | 7 | 300 | 64 | 1AE | No |
| 0.8 | 0.4 | 7 | 300 | 66 | 1AE | No |
| 0.4 | 0.8 | 7 | 300 | 66 | 1AE | No |
| 0.8 | 0.8 | 7 | 300 | 64 | 1AE | No |
| 0.4 | 0.4 | 9 | 300 | 66 | 1AE | No |
| 0.8 | 0.4 | 9 | 300 | 64 | 1AE | No |
| 0.4 | 0.8 | 9 | 300 | 64 | 1AE | No |
| 0.8 | 0.8 | 9 | 300 | 66 | 1AE | No |
| 0.2 | 0.6 | 8 | 200 | 65 | 1AE | No |
| 1 | 1 | 8 | 200 | 65 | 1AE | No |
| 0.6 | 0.2 | 8 | 200 | 65 | 1AE | No |
| 0.6 | 1 | 8 | 200 | 65 | 1AE | No |
| 0.6 | 0.6 | 6 | 200 | 65 | 1AE | No |
| 0.6 | 0.6 | 10 | 200 | 65 | 1AE | No |
| 0.6 | 0.6 | 8 | 0 | 65 | 1AE | No |
| 0.6 | 0.6 | 8 | 400 | 65 | 1AE | No |
| 0.6 | 0.6 | 8 | 200 | 63 | 1AE | No |
| 0.6 | 0.6 | 8 | 200 | 67 | 1AE | No |
| 0.6 | 0.6 | 8 | 200 | 65 | 1AE | No |
| 0.6 | 0.6 | 8 | 200 | 65 | 1AE | No |
| 0.6 | 0.6 | 8 | 200 | 65 | 1AE | No |
| 0.6 | 0.6 | 8 | 200 | 65 | 1AE | No |
| 0.6 | 0.6 | 8 | 200 | 65 | 1AE | No |
| 0.6 | 0.6 | 8 | 200 | 65 | 1AE | No |
| 0.4 | 0.4 | 7 | 100 | 66 | NE | No |
| 0.8 | 0.4 | 7 | 100 | 64 | NE | No |
| 0.4 | 0.8 | 7 | 100 | 64 | NE | No |
| 0.8 | 0.8 | 7 | 100 | 66 | NE | No |
| 0.4 | 0.4 | 9 | 100 | 64 | NE | No |
| 0.8 | 0.4 | 9 | 100 | 66 | NE | No |
| 0.4 | 0.8 | 9 | 100 | 66 | NE | No |
| 0.8 | 0.8 | 9 | 100 | 64 | NE | No |
| 0.4 | 0.4 | 7 | 300 | 64 | NE | No |
| 0.8 | 0.4 | 7 | 300 | 66 | NE | No |
| 0.4 | 0.8 | 7 | 300 | 66 | NE | No |
| 0.8 | 0.8 | 7 | 300 | 64 | NE | No |
| 0.4 | 0.4 | 9 | 300 | 66 | NE | No |
| 0.8 | 0.4 | 9 | 300 | 64 | NE | No |
| 0.4 | 0.8 | 9 | 300 | 64 | NE | No |
| 0.8 | 0.8 | 9 | 300 | 66 | NE | No |
| 0.2 | 0.6 | 8 | 200 | 65 | NE | No |
| 1 | 0.6 | 8 | 200 | 65 | NE | No |
| 0.6 | 0.2 | 8 | 200 | 65 | NE | No |
| 0.6 | 1 | 8 | 200 | 65 | NE | No |
| 0.6 | 0.6 | 6 | 200 | 65 | NE | No |
| 0.6 | 0.6 | 10 | 200 | 65 | NE | No |
| 0.6 | 0.6 | 8 | 0 | 65 | NE | No |
| 0.6 | 0.6 | 8 | 400 | 65 | NE | No |
| 0.6 | 0.6 | 8 | 200 | 63 | NE | No |
| 0.6 | 0.6 | 8 | 200 | 67 | NE | No |
| 0.6 | 0.6 | 8 | 200 | 65 | NE | No |
| 0.6 | 0.6 | 8 | 200 | 65 | NE | No |
| 0.6 | 0.6 | 8 | 200 | 65 | NE | No |
| 0.6 | 0.6 | 8 | 200 | 65 | NE | No |
| 0.6 | 0.6 | 8 | 200 | 65 | NE | No |
| 0.6 | 0.6 | 8 | 200 | 65 | NE | No |

**Table S2** - Sequences of primers and synthetic plasmid controls used.

| **ID** | **Sequence (5’ - 3’)** | **Observations/Ref.** |
| --- | --- | --- |
| N2_pUC57 | AACCCAAGGAAATTTTGGGGACCAGGAACTAATCAGACAAGGAACTGATTACAAACATTGGCCGCAAATTGCACAATTTGCCCCCAGCGCTTCAGCGTTCTTCGGAATGTCGCGCATTGGCATGGAAGTCACACCTTCGGGAACGTGGTTGACCTACACAGGTGCCATCAAATTGGATGACAAAGATCCAAATTTCAAAGATCAAGTCATTTTGCTGAATAAGCATAT | Synthetic control sequence cloned into the EcoRV site of pUC57 |
| Orf1ab_pUC57 | CAAGTATTTTAGTGGAGCAATGGATACAACTAGCTACAGAGAAGCTGCTTGTTGTCATCTCGCAAAGGCTCTCAATGACTTCAGTAACTCAGGTTCTGATGTTCTTTACCAACCACCACAAACCTCTATCACCTCAGCTGTTTTGCAGAGTGGTTTTAGAAAAATGGCATTCCCATCTGGTAAAGTTGAGGGTTGTATGGTACAAGTAACTTGTGGTACAACTACACTTAACGGTCTTTGGCTTGATGACGTAGTTTACTGTC | Synthetic control sequence cloned into the EcoRV site of pUC57 |
| E1_pUC57 | ATGTACTCATTCGTTTCGGAAGAGACAGGTACGTTAATAGTTAATAGCGTACTTCTTTTTCTTGCTTTCGTGGTATTCTTGCTAGTTACACTAGCCATCCTTACTGCGCTTCGATTGTGTGCGTACTGCTGCAATATTGTTAACGTGAGTCTTGTAAAACCTTCTTTTTACGTTTACTCTCGTGTTAAAAATCTGAATTCTTCTAGAGTTCCTGATCTTCTGGTCTAA | Synthetic control sequence cloned into the EcoRV site of pUC57 |
| N2-F3 | ACCAGGAACTAATCAGACAAG | doi.org/10.2144/btn-2020-0078 |
| N2-B3 | GACTTGATCTTTGAAATTTGGATCT | doi.org/10.2144/btn-2020-0078 |
| N2-FIP | TTCCGAAGAACGCTGAAGCGGAACTGATTACAAACATTGGCC | doi.org/10.2144/btn-2020-0078 |
| N2-BIP | CGCATTGGCATGGAAGTCACAATTTGATGGCACCTGTGTA | doi.org/10.2144/btn-2020-0078 |
| N2-LF | GGGGGCAAATTGTGCAATTTG | doi.org/10.2144/btn-2020-0078 |
| N2-LB | CTTCGGGAACGTGGTTGACC | doi.org/10.2144/btn-2020-0078 |
| Orf1ab - F3 | TGGATACAACTAGCTACAGAGAAG | doi.org/10.1038/s41598-021-84792-8 |
| Orf1ab - B3 | AGCCAAAGACCGTTAAGTGTA | doi.org/10.1038/s41598-021-84792-8 |
| Orf1ab - FIP | GTGGTGGTTGGTAAAGAACATCAGACTTGTTGTCATCTCGCAAAGG | doi.org/10.1038/s41598-021-84792-8 |
| Orf1ab - BIP | CCTCTATCACCTCAGCTGTTTTGCTGTACCATACAACCCTCAACTT | doi.org/10.1038/s41598-021-84792-8 |
| Orf1ab - LF | ACCTGAGTTACTGAAGTCATTGAGA | doi.org/10.1038/s41598-021-84792-8 |
| Orf1ab - LB | TGGTTTTAGAAAAATGGCATTCCC | doi.org/10.1038/s41598-021-84792-8 |
| E1_F3 | TGAGTACGAACTTATGTACTCAT | doi.org/10.2144/btn-2020-0078 |
| E1_B3 | TTCAGATTTTTAACACGAGAGT | doi.org/10.2144/btn-2020-0078 |
| E1_FIP | ACCACGAAAGCAAGAAAAAGAAGTTCGTTTCGGAAGAGACAG | doi.org/10.2144/btn-2020-0078 |
| E1_BIP | TTGCTAGTTACACTAGCCATCCTTAGGTTTTACAAGACTCACGT | doi.org/10.2144/btn-2020-0078 |
| E1_LF | CGCTATTAACTATTAACG | doi.org/10.2144/btn-2020-0078 |
| E1_LB | GCGCTTCGATTGTGTGCGT | doi.org/10.2144/btn-2020-0078 |
| ACTB - F3 | AGTACCCCATCGAGCACG | doi.org/10.2144/btn-2020-0078 |
| ACTB - B3 | AGCCTGGATAGCAACGTACA | doi.org/10.2144/btn-2020-0078 |
| ACTB - FIP | GAGCCACACGCAGCTCATTGTATCACCAACTGGGACGACA | doi.org/10.2144/btn-2020-0078 |
| ACTB - BIP | CTGAACCCCAAGGCCAACCGGCTGGGGTGTTGAAGGTC | doi.org/10.2144/btn-2020-0078 |
| ACTB - LF | TGTGGTGCCAGATTTTCTCCA | doi.org/10.2144/btn-2020-0078 |
| ACTB - LB | CGAGAAGATGACCCAGATCATGT | doi.org/10.2144/btn-2020-0078 |

**Table S3** - Reaction parameters of sample solutions with different pHs. TTR and Nmax are expressed as averages of three replicates.

| **Sample Solution pH** | **TTR (s)** | **Nmax (delta)** |
| --- | --- | --- |
| 7.0 | 647 ± 53 | 68 |
| 7.5 | 716 ± 25 | 72 |
| 8.0 | 946 ± 175 | 73 |
| 8.5 | 1267 ± 438 | 79 |
| 9.0 | 837 ± 77 | 77 |


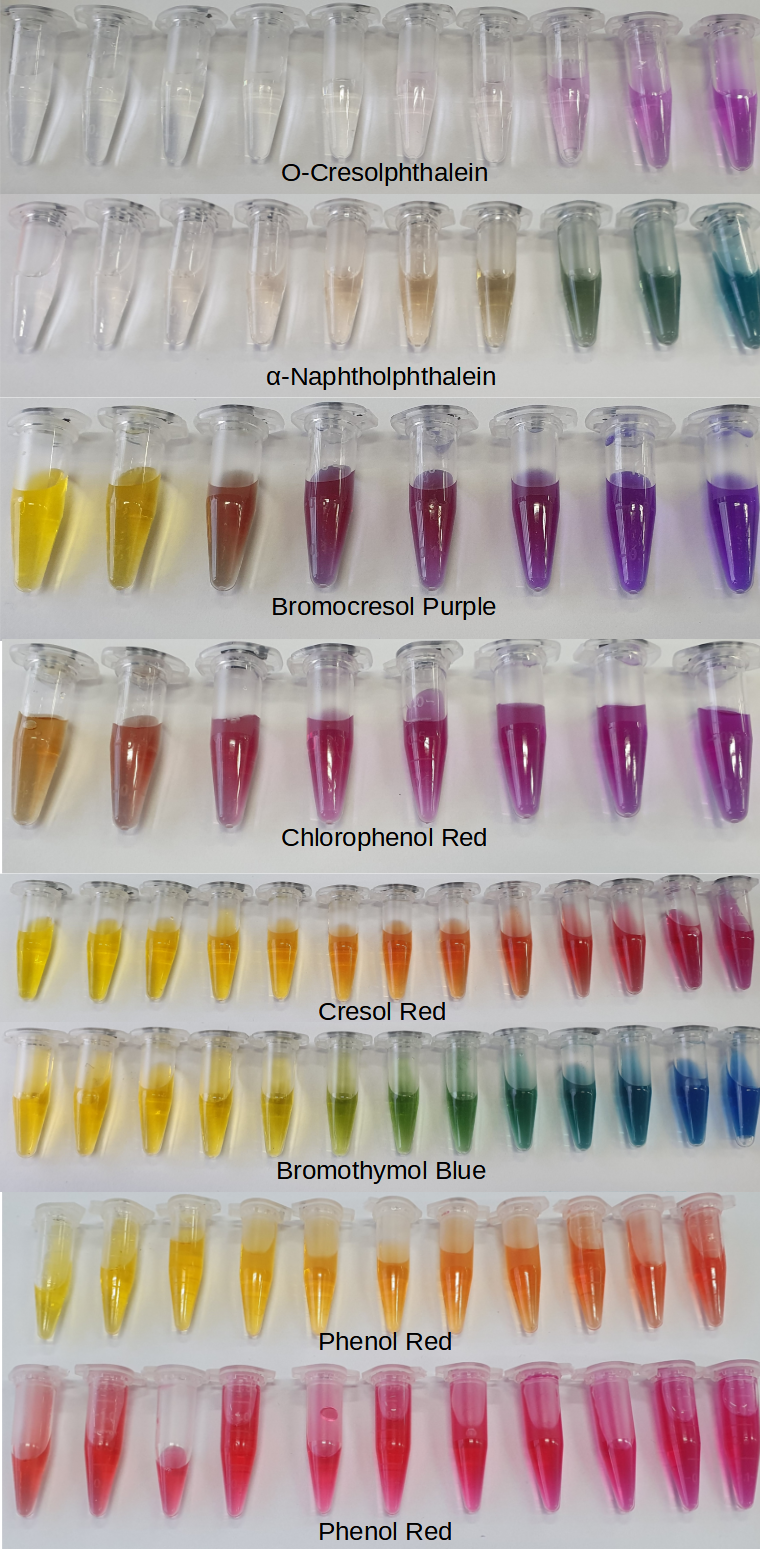


**Figure S1 -** Color gradient of each pH indicator dye tested. The pH range was approximately between 4 to 10. Each dye has a different pH interval.

**
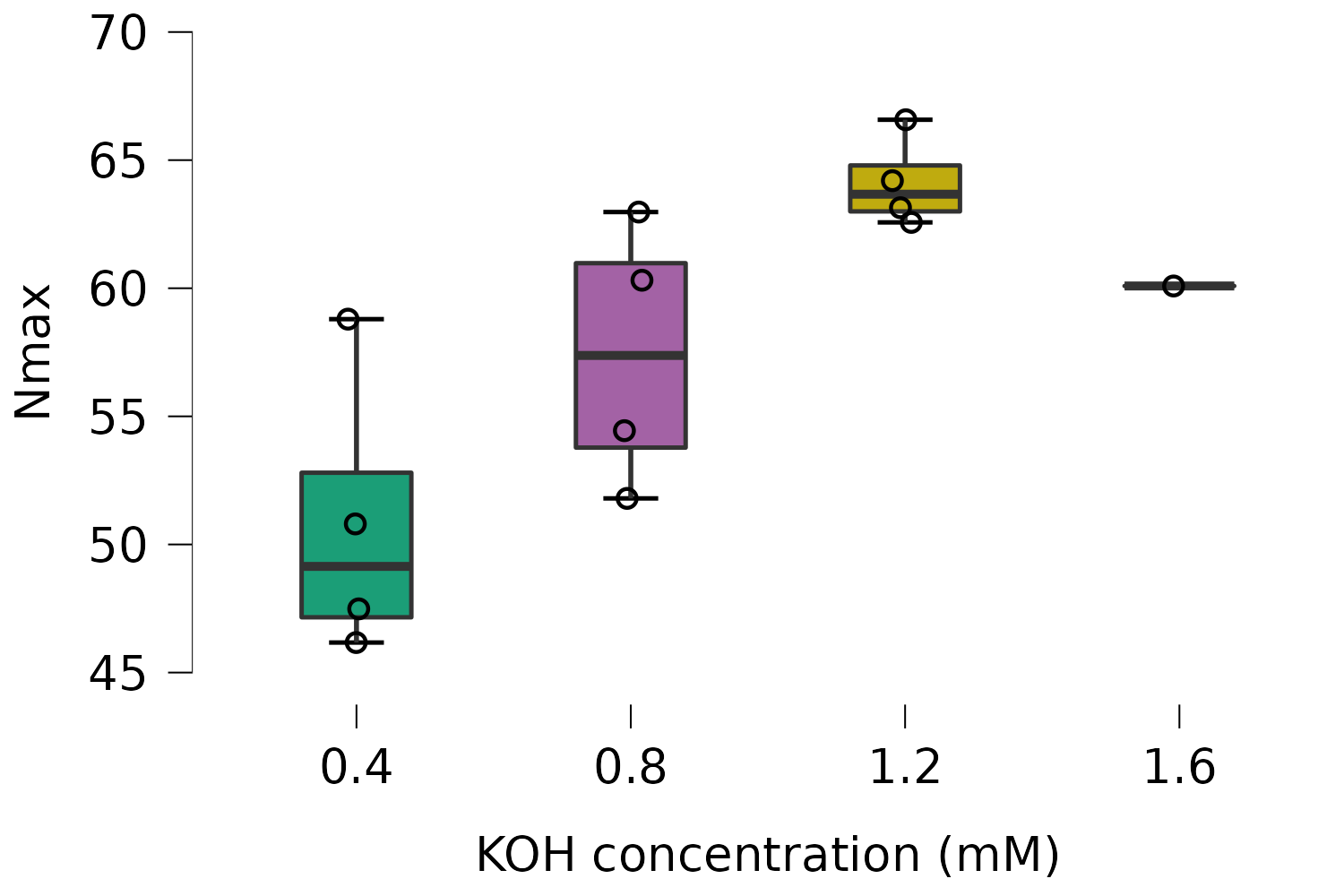
A**


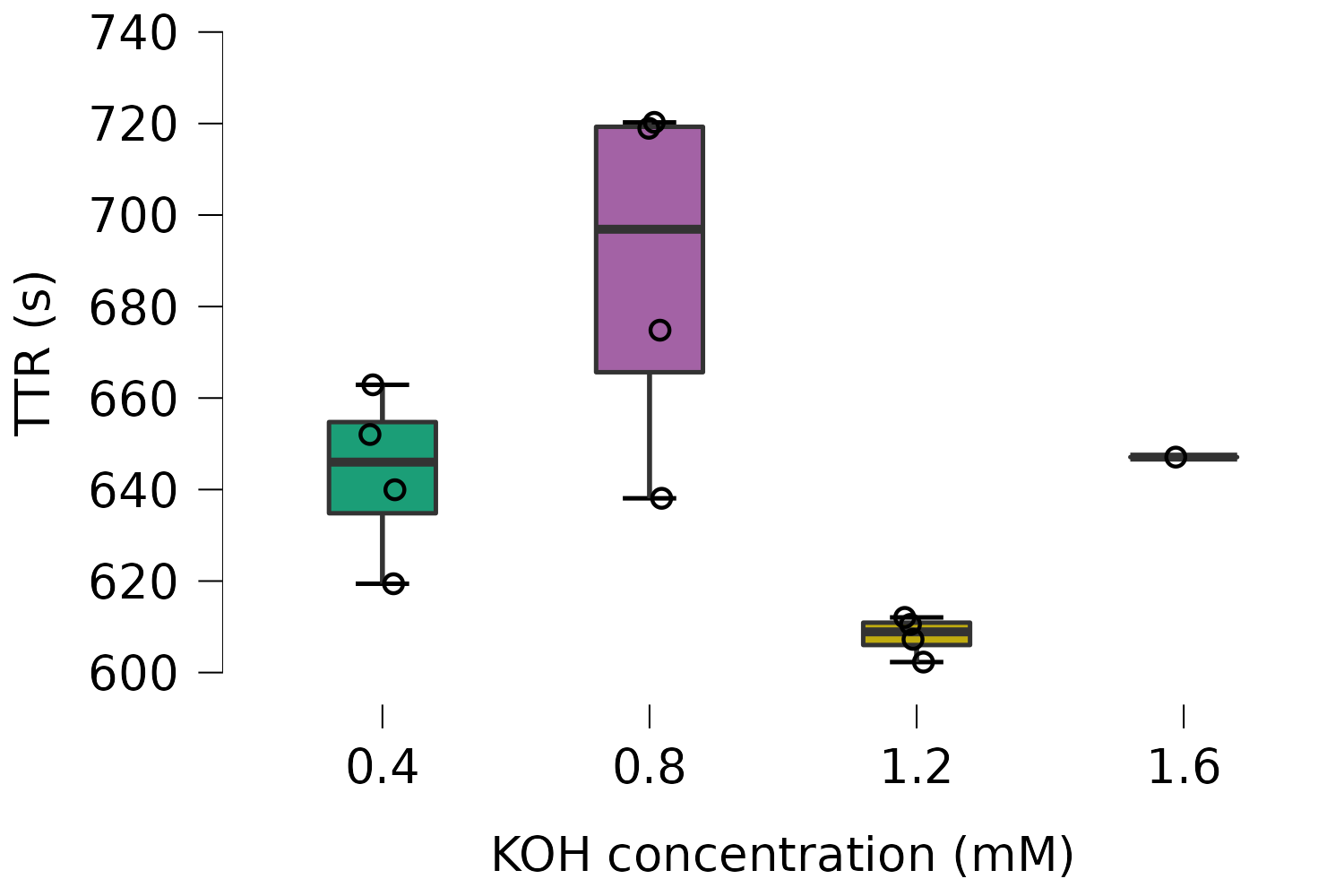
**B**

**Figure S2 -** Effect of different KOH concentrations (mM) (x-axis) in the Nmax (**A**) and in the TTR of the reaction (**B**). No statistically significant difference was seen.

**
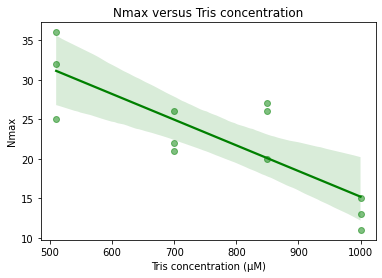
A**

**
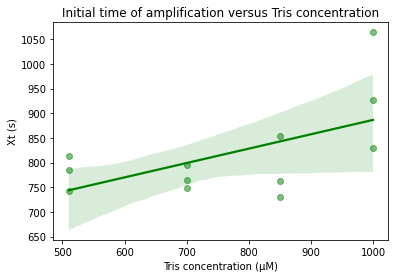
B**

**Figure S3 -** Effect of different Tris concentrations (510, 700, 850 and 1000 μM) (x-axis) in the Nmax (**A**) and in the initial time of amplification (Xt) of the reaction (**B**).


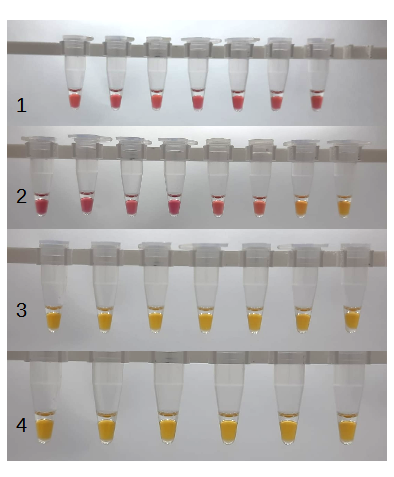


**Figure S4 -** Tubes with Orf1ab and N primers after 30-minute amplification at 67 °C. The numbers of genomic equivalent copies are 1) 10 copies/reaction; 2) 50 copies/reaction; 3) 100 copies/reaction; 4) 1000 copies/reaction. 100 genomic copies/reaction had 100% positivity. Each reaction had a final volume of 20 μL.
